# Predictors of Spasticity After Stroke

**DOI:** 10.1007/s40141-016-0128-3

**Published:** 2016-07-22

**Authors:** Katharina Stibrant Sunnerhagen

**Affiliations:** Department of Rehabilitation Medicine, Institute of Neuroscience and Physiology, University of Gothenburg, Gothenburg, Sweden

**Keywords:** Poststroke spasticity, Stroke rehabilitation, PSS, Risk factors, Outcome, Review

## Abstract

**Purpose of Review:**

Spasticity is a part of the upper motor neuron syndrome and can result in reduced function. Reduction of the complications may be facilitated by early intervention, making identification of stroke patients at high risk for developing spasticity essential.

**Recent Finding:**

Different predictors of poststroke spasticity (PSS) have been suggested in different studies, including development of increased muscle tone, greater severity of paresis, sensory impairment, and low Barthel Index score. The results also indicate that early identification of factors predictive of PSS is beneficial.

**Summary:**

In this review article, the results of five studies are discussed and they all support the notion that early identification of factors predictive of PSS is beneficial and could help to identify individuals who would benefit most from intervention and thereby provide better outcome.

## Introduction

Spasticity is a part of the upper motor neuron syndrome and can result in reduced function. The impact of spasticity on poststroke recovery may not be obvious at first, and hence not addressed in the early phase. Spasticity in the upper limb spasticity has been found to be associated with reduced arm function and lower levels of independence, and with an astounding four-fold increase in direct care costs during the first-year poststroke [1, 2]. It seems as if spasticity in patients with stroke is more common in the upper extremity than in the lower limbs [3•], The frequency of spasticity in the upper limb varies from 7 % to 38 % [2, 4–6] in the first 12 months, and was found to be 46 % in patients with initial impaired arm function [7].

Early recognition of spasticity, and identification of predictors to assist rehabilitation professionals recognize which stroke patients are at risk for spasticity to develop, is suggested to yield earlier treatment of poststroke spasticity (PSS) and possibly better outcomes [8]. It would be helpful to know which factors can identify patients are at high risk of developing severe (PSS) especially during the initial admission poststroke. Increased knowledge regarding the pathophysiology, evolution of the condition, epidemiology, and therapeutic intervention for PSS, along with identifying PSS risk factors, is a road for better care [9].

The aim of this review is to present predictors that will help to identify which patients are at risk for the development of PSS.

## Materials and Methods

A search was performed using the following terms: natural history OR clinical course OR prevalence AND stroke AND spasticity, with the limitation to publications written in English. The results were further limited to studies focusing on the prediction of spasticity (up to 12 months poststroke).

Various studies have assessed spasticity at various time points poststroke, (Table 1). Although a time lag may exist between stroke onset and the onset of PSS, this has only been addressed in a few studies. In a classic study from Sommerfeld et al. [10], at baseline (mean: 5.4 days), 21 % of patients with first-ever stroke were deemed to have spasticity according to the Modified Ashworth Scale (MAS score >0), while 19 % demonstrated spasticity at 3 months poststroke. Patients with disabling spasticity score significantly worse than those with mild spasticity on the modified Rankin Scale (mRS *P* = 0.009) and the Barthel Index (BI *P* = 0.005), suggest that treatment needs are not the same for all patients with spasticity, and those with disabling spasticity must be distinguished from those with less serious deficits [2].

**Table 1:** Poststroke spasticity in patients admitted to a stroke unit

Study	N	Time after stroke	Evaluation method	Predictors of spasticity	Prevalence of spasticity
Opheim A et al. (2014)	117	Up to 12 months	MAS	Reduced sensorimotor function	Spasticity at 3 and 10 days and 4 weeks, 24, 43, and 46 % of. At 12 months, 46 %
				Reduced sensation	
Wissel J et al. (2010)	94	Up 10 4 months	Modified ashworth scale (Spasticity: MAS > 0)	Moderate increase in muscle tone at baseline and/or first follow-up (MAS = 2), low Barthel Index at baseline, hemispasticity, involvement of more than two joints at first follow-up, and paresis at any assessment point	Spasticity: At 2 weeks: 24.5 % At 6 weeks: 26.7 % At 4 months: 21.7 % Severe spasticity: 9.6 % (MAS ≥ 3)
Urban PP et al. (2010)	211	Up to 6 months	Modified ashworth scale (Spasticity: MAS > 0)	More severe paresis in the proximal and distal limb muscles had a higher risk for developing spasticity	42.6 % had developed spasticity at 6 months. A more severe degree of spasticity (Modified Ashworth Scale > or = 3) was observed in 15.6 % of all patients

## Predictors of Spasticity Development

Not much has been published regarding the onset and course of spasticity during the first-year poststroke. Although the prediction of late-developing PSS is likely improved using measures of early PSS, the time point at which these measures should be taken is unclear. Different studies have shown some similar and some varied results.

Lundstrom et al. [3] included 49 subjects with any paresis after first-ever stroke and examined them at baseline (day 2–10 after stroke) and at 2 follow-up time points, at 1 and 6 months. At all time occasions, muscle tone (assessed by MAS), global disability (assessed by the mRS), stroke severity, paresis severity, and sensory disturbance (all assessed by the National Institutes of Health Stroke Scale [NIHSS]) were recorded to determine the occurrence of and risk factors for PSS. At baseline, 4 % (2 of 49) of patients had PSS (MAS score ≥1). Early PSS development was further observed in 27 % (13 of 48) of patients at 1 month and 23 % (11 of 47) at 6 months. Spasticity in the upper limb was observed more often than in the lower limb: at 1 month, 25 % of patients had PSS in the upper limb versus 13 % in the lower limb, and at 6 months the rates were 22 and 13 %, respectively. Disabling spasticity was observed in 13 % (6 of 47) of patients at the 6-month follow-up [3]. Multiple logistic regression analysis found severe paresis of the arm (>2 points on item 5 of the NIHSS) at baseline to be associated with a higher risk for spasticity (*P* < 0.001) as early as 1 month after stroke (Table 2). Smoking and younger age was also associated with increased risk. The six patients displaying disabling spasticity throughout the follow-up period had initial severe arm paresis as well (*P* < 0.002).

**Table 2:** Risk factors significantly predictive of permanent poststroke spasticity [6•]

Risk factor	*P* value
Any paresis in affected limb	<0.001
MAS ≥2 in ≥1 joint within median 6 weeks poststroke	0.01
>2 joints affected by increased muscle tone	0.002
Hemispasticity within median 6 weeks poststroke	0.01
Lower Barthel Index score at baseline	0.002
More severe paresis at median 16 weeks poststroke	0.02

A larger study [5•] of 301 subjects with first-ever stroke and limb paresis were included and examined in the acute phase after stroke (within 5 days) and again 6 months later. At both time points, the degree and pattern of paresis and muscle tone (assessed via the British Medical Research Council scale and MAS) as well as Barthel Index were evaluated [5•]. Of the 211 subjects reassessed at 6 months, 42.6 % (*n* = 90) had developed PSS (MAS score ≥1). A more severe degree of spasticity (MAS score ≥3) was observed in 15.6 % (*n* = 33) of these subjects. Logistic regression analysis confirmed that subjects with a more severe degree of paresis (BMRC grades 1 and 0) in the proximal and distal muscles of the upper and lower limbs during the acute stage had a higher risk of developing PSS (*P* ≤ 0.001). Furthermore, the development of PSS in the upper and lower limbs was more frequent in subjects with initial hemihypesthesia than in subjects without sensory deficits (OR = 2.27; *P* = 0.011). However, the sensory examination included only sensitivity to light touch. The conclusion was that predictors for the development of PSS included a severe degree of paresis and hemihypesthesia at stroke onset. Lower BI at 6 months, were more common in subjects with PSS (*P* < 0.001) compared to the group without PSS at 6 months [5•].^8^

Wissel and coworkers [6•] presented a study of 94 subjects without preexisting spasticity who were examined at 6 days poststroke (baseline; range 1–14 days), 6 weeks (range 4–12 weeks), and 16 weeks (range 12–24 weeks). At all time points, muscle tone (MAS), pain, paresis, and BI score were recorded to identify risk factors for the development of PSS. Similar to other studies, early development of PSS was observed, with 24.5 % (23 of 94) of subjects with stroke developing an increase in muscle tone (MAS score >0) within 2 weeks after stroke [6•]. Spasticity was observed in 26.7 % (23 of 86) of patients by the first follow-up at 6 weeks after stroke and in 21.7 % (18 of 83) by 16 weeks after stroke. Among all subjects who developed PSS at any time during the course of the study, 98 % exhibited PSS by the first follow-up (median of 6 weeks). Subjects with PSS had significantly higher incidences of pain (*P* < 0.001) and nursing home placement (*P* < 0.05) and lower BI (*P* = 0.035) scores than subjects with normal muscle tone [6•]. Predictors of the development of permanent spasticity are shown in Table 2. Severe spasticity (MAS score ≥3) was predicted by lower BI scores (median score 63.3, compared to 80.6 in patients without spasticity) at baseline and moderate muscle tone (MAS score = 2) at baseline to 6 weeks poststroke. Presence of hemiparesis correlated with PSS at first (*P* = 0.02) and second (*P* = 0.005) follow-up. Hemispasticity and increased muscle tone (MAS score ≥1) in more than two joints at 6 weeks, and more severe degrees of paresis at 16 weeks, were risk factors for permanent spasticity. Severity of paresis at the second follow-up (12–24 weeks after stroke) significantly correlated with degree of spasticity (*P* = 0.02). Lower BI at baseline predicted development of more severe spasticity at final follow-up (*P* = 0.002). None of the baseline characteristics, such as age, sex, location or type of stroke, or smoking, was significantly associated with the development of PSS in this sample [6•].

Another study [11] investigated patients admitted to a rehabilitation unit after a first-ever ischemic stroke. (*N* = 163) Again, MAS was used for assessing upper limb spasticity. The upper limb function was assessed and for ADL, the Barthes Index was used. Upper limb spasticity was defined as MAS of 1 or greater. In this sample [11], upper limb spasticity occurred in 1/3 of the patients (54 patients) at 3 months after stroke. Development of spasticity at later stages of the stroke was infrequent, occurring in only 28 patients (17 %). However, in almost half of those with moderate spasticity (MAS 2) at 3 months, the spasticity progressed to severe (MAS 3). As predictors, poor upper limb function was the most important factor for “moderate to severe spasticity” (MAS ≥2) (*P* < .001) [11],

A recent study [12•] which included 117 patients who had experienced a stroke for the first time and had a documented upper extremity paresis day 3 poststroke were consecutively included from a comprehensive stroke unit. Evaluations were made at admission and at 3 and 10 days, 4 weeks and 12 months poststroke. Upper limb spasticity in elbow flexion/extension and wrist flexion/extension was assessed (at all time points except admission) with modified Ashworth scale (MAS). “Any spasticity” was regarded as MAS ≥1, and “severe spasticity” was regarded as MAS ≥2 in any of the muscles. Fugl-Meyer assessment scale was used to assess sensorimotor function, sensation, pain and joint range of motion in the upper limb. The results from this scale together with demographic and diagnostic information were included in both univariate and multivariate logistic regression analysis models. Due to the long follow-up there were dropouts and in the logistic regression analysis, data from 76 patients were included [12•]. The results indicated that sensorimotor function was the most important predictor both for “any” and “severe spasticity” 12 months poststroke. In addition, spasticity 4 weeks poststroke was a significant predictor for “severe spasticity.” The best prediction model for “any spasticity” was observed 10 days poststroke (85 % sensitivity, 90 % specificity). The best prediction model for “severe spasticity” was observed 4 weeks poststroke (91 % sensitivity, 92 % specificity) [12•].

## Conclusion

Despite evidence supporting the possibility that these additional predictive factors may be of clinical importance—particularly motor impairment, neurologic and sensory deficit, associated diseases, and surrogate markers from magnetic resonance imaging such as lesion volume and location—further investigation is warranted in order to validate their correlation with PSS development.

Further research to determine the clinical utility of models to predict the onset of PSS and other common poststroke complications is also warranted. Additionally, research to determine the specificity of how upper and lower limb spasticity contributes to the outcome and quality of life would be welcome to this field. These findings point out that individualized assessment of different locations and degrees of spasticity and paresis need to be carefully documented before starting any treatment.
